# 

**Published:** 1855-04

**Authors:** 


### RECEIPTS FOR THE REGISTER.

| | | | |
|---|---|---|---|
| Dr. R. E. Cole, Cal | 4 00 | Dr. C. Bonsall, | 4 00 |
| Dr. C. E. Kells, N. O | 2 00 | Dr. W. H. Goddard, | 2,00 |
| Dr. A. J. Reeve, | 1 50 | Dr. H N. Lewis, | 12 00 |
| Dr. J. M. Shepherd | 5 00 | Dr. D. Walters, | 4 00 |
| Dr. E. Stone, | 2 00 | Drs. L. D. & J. S. Walters, | 2 00 |
| Dr. J. W. Keely | 2 00 | Dr. J. D. Thompson, | 1 00 |
| Dr. B. A. Satterthwaite, | 2 00 | Dr. E. Bray, | 2 00 |
| Dr. R. M. Kenedy, | 2 00 | Dr. J. Hood, | 4 00 |
| Dr. J. Sanford, | 3 00 | Dr. S. P. Miller, | 4 00 |
| Dr. T. McAboy, | 2 00 | Dr. S. Clippenger, | 5 00 |
| Dr. F. M. Perry, | 5 00 | Dr. M. C. Browning,- | 8 00 |
| Dr. B. Strickland, | 12 00 | Dr. J. H. Farnsworth, | 10 00 |
| Dr. H. Fenn, | 4 00 | Dr. J. Douthett, | 10 50 |
| Dr. A. W. Newton, | 5 00 | Dr. J. C. Whinery, | 4 00 |
| Dr. J. Harris | 2 00 | Dr. W. Potter, | 4 00 |
| Dr. W. R. Webster, | 2 50 | Dr. J. H. Hyde, | 2 00 |
| Dr. M. DeCamp, | 2 00 | Dr. W. A. Pease, | 2 00 |
| Dr. J. P. Ulrey, | 4 00 | Dr. E. C. Sloan, | 2 00 |
| Dr. C. H. Quinlan, | 4 00 | Dr. J. G. Ambler, | 8 25 |
| Dr. E. Townsend, | 8 00 | Dr. J. P. Lucas, | 4 00 |

### THE OHIO COLLEGE OF DENTAL SURGERY.

### Session of 1855–'56.

The regular course of lectures in this institution, commences on the first Monday of November, and closes on Tuesday, succeeding the third Monday of February.

### FACULTY.

JAMES TAYLOR, M. D., D. D. S.
*Professor of the Institutes of Dental Science,*

C. B. CHAPMAN, M. D.,
*Professor of Anatomy and Physiology.*

J. B. SMITH, M. D.,
*Professor of Pathology and Therapeutics.*

JONATHAN TAFT, D. D. S.,
*Professor of Operative Dentistry.*

H. R. SMITH, D. D. S.,
*Professor of Mechanical Dentistry.*

GEORGE WATT, M. D., D. D. S.,
*Professor of Chemistry and Metallurgy.*

The Infirmary will be kept open most of the summer, and during the month of October special attention will be paid to operations before such of the class as can come in by that time. Those desiring *extra* opportunities for practice should avail themselves of this months privileges.

To meet the wants of those engaged in practice, half course tickets are issued, thus enabling them to make out a full course for graduation without leaving their homes and practice for one entire session.

Students should bring with them such instruments as they may have on hand, also the following books, which are more or less used as Text Books: Harris' Principles and Practice of Dental Surgery, Jourdain's Surgical Diseases of the Mouth, Wilson's Anatomy, Carpenter's Physiology, Dunglison's Therapeutics, and Turner's Chemistry.

Candidates for graduation must have attended two full courses of Lectures, the last in this school. Certificates of four years Dental Practice, or of one full course in any respectable Dental or regular Medical College, will be received as equivalent to the first course.

### TERMS OF ADMISSION.

Tickets are considered as cash, and when not paid for in advance, a note to the Faculty, with approved security, will be required.

| | |
|---|---|
| Tickets for the entire course, and admissions to dissections, | $100 00 |
| Marticulation Ticket, - - - - - - - | 5 00 |
| Diploma Fee, - - - - - - - | 25 00 |

Good and respectable Board can be had a from $2,50 to $3,00 per week.

JAMES TAYLOR, Dean.

